# POU6F2, a risk factor for glaucoma, myopia and dyslexia, labels specific populations of retinal ganglion cells

**DOI:** 10.1038/s41598-024-60444-5

**Published:** 2024-05-02

**Authors:** Fangyu Lin, Ying Li, Jiaxing Wang, Sandra Jardines, Rebecca King, Micah A. Chrenek, Janey L. Wiggs, Jeffrey H. Boatright, Eldon E. Geisert

**Affiliations:** 1 Department of Ophthalmology, Emory University, 1365B Clifton Road NE, Atlanta, GA 30322 USA; 2 Massachusetts Eye and Ear, Harvard Medical School Boston, Boston, MA USA; 3 Atlanta Veterans Administration Center for Visual and Neurocognitive Rehabilitation, Decatur, GA USA; 4 Present Address: Icahn School of Medicine at Mount Sinai, 1 Gustave L. Levy Pl, New York, NY 10029 USA

**Keywords:** POU6F2, Cdh6, Hoxd10, Retinal ganglion cell, Glaucoma, Dyslexia, Myopia, ON–OFF directionally selective, Optic nerve crush, Mouse, CART, DBA/2J, Visual system, Retina, Eye diseases

## Abstract

*Pou6f2* is a genetic connection between central corneal thickness (CCT) in the mouse and a risk factor for developing primary open-angle glaucoma. *POU6F2* is also a risk factor for several conditions in humans, including glaucoma, myopia, and dyslexia. Recent findings demonstrate that POU6F2-positive retinal ganglion cells (RGCs) comprise a number of RGC subtypes in the mouse, some of which also co-stain for Cdh6 and Hoxd10. These POU6F2-positive RGCs appear to be novel of ON–OFF directionally selective ganglion cells (ooDSGCs) that do not co-stain with CART or SATB2 (typical ooDSGCs markers). These POU6F2-positive cells are sensitive to damage caused by elevated intraocular pressure. In the DBA/2J mouse glaucoma model, heavily-labeled POU6F2 RGCs decrease by 73% at 8 months of age compared to only 22% loss of total RGCs (labeled with RBPMS). Additionally, *Pou6f2*^*−/−*^ mice suffer a significant loss of acuity and spatial contrast sensitivity along with an 11.4% loss of total RGCs. In the rhesus macaque retina, POU6F2 labels the large parasol ganglion cells that form the magnocellular (M) pathway. The association of POU6F2 with the M-pathway may reveal in part its role in human glaucoma, myopia, and dyslexia.

## Introduction

POU6F2 was initially identified as a novel POU-domain transcription factor within a subpopulation of RGCs in the retina of mice, cats, and monkeys^1^. Subsequently, we discovered that *Pou6f2* is a genomic element that modulates central corneal thickness (CCT), an ocular trait that may influence the development of glaucoma, as defined in the Ocular Hypertension Treatment Studies (OHTS)^2–4^. Thinner corneas are associated with a greater risk of developing primary open-angle glaucoma (POAG), independent of the confounding effects of CCT on intraocular pressure measurements^2,4^. We confirmed that POU6F2 is expressed in a subset of adult mouse RGCs, similar to previous findings^1^, and that this population of RGCs is susceptible to glaucomatous injury^5^.

The identification of mouse RGC subtypes was facilitated by recent advances in single-cell RNA sequencing and transcriptional profiling. This unbiased quantitative approach used to cluster RGCs based on similar gene expression profiles has provided a more accurate understanding of RGC subtypes in the mouse retina^6,7^. Tran et al.^7^ conducted a study on RGCs from adult mice and identified six RGC subtypes that express high levels of *Pou6f2*. These six subtypes were all classified as "Novel": C7-Novel, C8-Novel, C10-Novel, C18-Novel, C37-Novel, and C44-Novel. Additionally, there was another subset of *Pou6f2*-positive cells that expressed low levels of the mRNA.

The present study focuses on characterizing POU6F2-positive RGC subtypes and defining the role of POU6F2 in visually-guided behavior using the *Pou6f2*^*−/−*^ mouse model. This mouse exhibits a loss of RGCs and a significant decrease in visual acuity and contrast sensitivity compared to wild-type littermates. Since these early investigations, several genome-wide association studies (GWAS) have implicated POU6F2 as a potential risk factor for human eye diseases, including glaucoma^8–10^, myopia^11^, and dyslexia^12^. Translating the findings in the mouse retina to humans is complicated by the dramatic difference between mouse RGCs and human RGCs. To provide a better understanding of the role of POU6F2 in human retina, the current study was undertaken to define the RCG subtypes in the macaque retina that express POU6F2. Our findings in the macaque retina shed light on the role of POU6F2 in human glaucoma, myopia and dyslexia.

## Results

### Morphological characterization of POU6F2 RGCs

In flat-mounts of the C57BL/6J mouse retina, POU6F2 labeled nuclei of cells that were also positive for RBPMS and THY1 (pan-RGC markers, Table 1). The intensity of the POU6F2 labeling varied considerably. Some RGCs have highly labeled nuclei while others have lightly labeled nuclei (Supplemental Fig. 1). We categorized the cells into heavily labeled and lightly labeled POU6F2 RGCs. The lightly labeled cells had less than 50% of the fluorescent intensity of the heavily labeled RGCs. In the RGC layer of the C57BL/6J mouse, approximately 15.2% of the RBPMS-positive RGCs were heavily labeled with POU6F2. The remaining POU6F2-positive cells were moderately to lightly labeled and represented 17.2% of the RGCs. There was a modest number of POU6F2-positive cells in the inner surface of the inner nuclear layer, suggestive of amacrine cell labeling. However, the POU6F2-positive cells at the surface of the inner nuclear layer were also positive for the RGC marker RBPMS, indicating that these cells may be displaced RGCs. To determine if the POU6F2 cells were all RGCs, we crushed the optic nerve and examined retinas 28 days after crush. All of the nuclear staining in the ganglion cell layer and in the amacrine cell layer was lost. These additional data extend our finding from previous studies^5^.

**Table 1:** Staining of retinal cells with cell type specific markers.

Marker	Marker	Strain	Co-staining
THY1	Pan-RGCs	Thy1-YFP-H	100%*
RBPMS	Pan-RGCs	C57BL/6J	100%*
TUJ1	Pan-RGCs	C57BL/6J	100%*
CART	ooDSGCs	C57BL/6J	No
SATB2	ooDSGCs	C57BL/6J	No
CDH6	ooDSGCs	Cdh6-CreER	24%^†^
HOXD10	ooDSGCs	Hoxd10-GFP	100%^†^
SMI32	Alpha RGCs	C57BL/6J	No
ChAT	Amacrine	C57BL/6J	No
GAD67	Amacrine	C57BL/6J	No

*Co-staining represents the percentage of POU6F2-positive cells in the total population of the marker-positive cells.

^†^Represents the percentage of Cdh6-positive RGCs and Hoxd10-positive RGCs co-that are stained with POU6F2.

**Figure 1:**
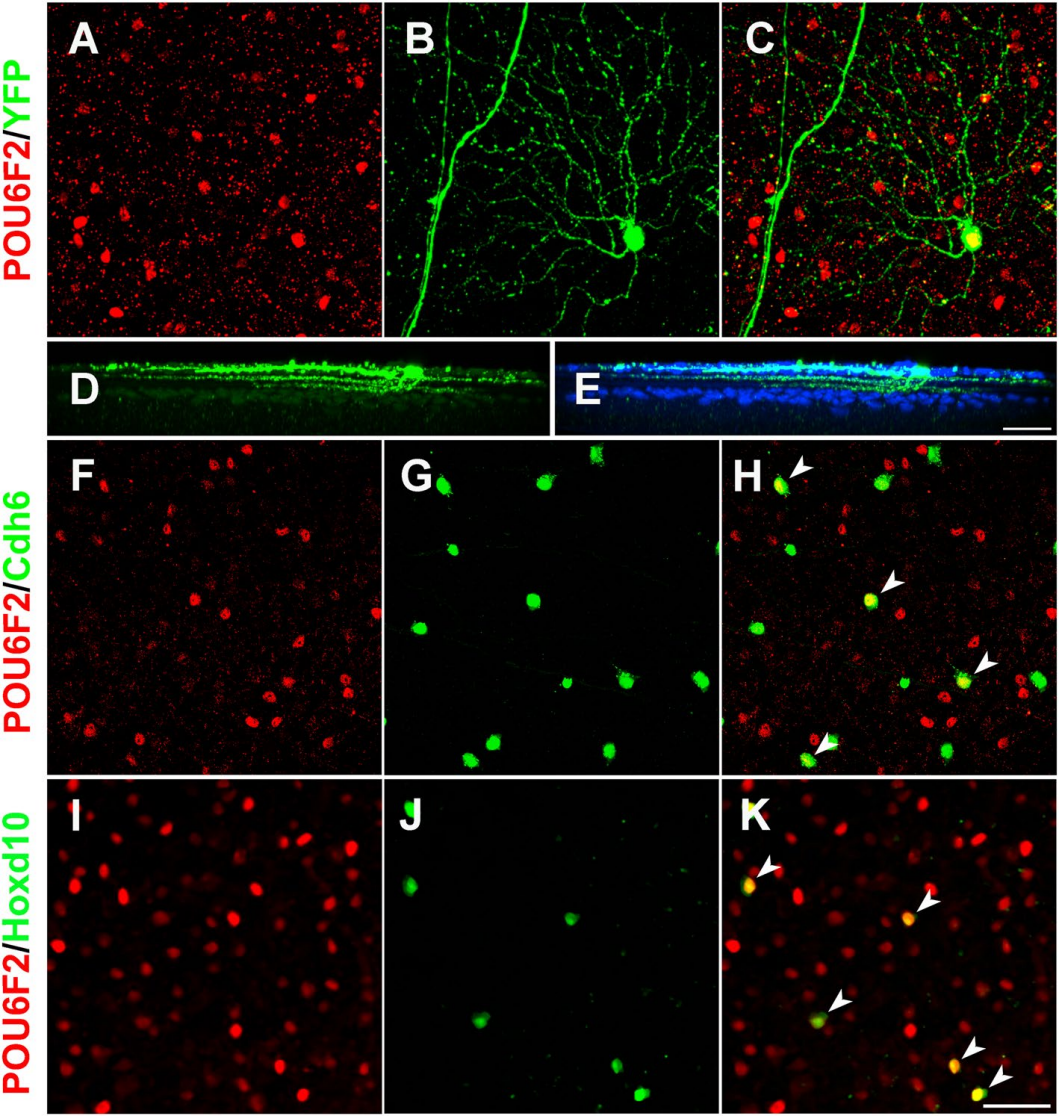
Identification of POU6F2 positive RGC subtypes. Dendritic morphology of heavily labeled POU6F2 cells indicates they are ON–OFF RGCs (**A-D**). The retinas of Thy1-YFP-H mice were stained for POU6F2 (**A**) and GFP (**B**). The merged channel (**C**) reveals the POU6F2-positive nucleus (Red in **A**) can be seen in the YFP labeled RGC (Green). A 90-degree rotation of the 3D reconstruction is shown (**D**) reveal the bistratified dendrites in the inner plexiform layer. Amacrine cells were labeled with ChAT (Blue in **E**) mark the location of sublaminae S2 and S4. Notice the distribution of labeled dendrites in the ON and OFF sublaminae showing the POU6F2 RGC is bistratified ON–OFF RGC. Retinal flat mounts of the *Cdh6*-CreER mice were stained for POU6F2 (**F–H**). Of the Cdh6-positive RGCs (**G**) 24% were also positive for POU6F2 (**F**) forming a distinct subpopulation of the POU6F2-positive RGCs (arrow heads in **H**). Retinal flat-mounts of the Hoxd10-YFP retinas were stained for POU6F2 (**I–K**). In a merged image (**K**), all of the Hoxd10 positive cells were also positive for POU6F2 (arrow heads). Scale bar in E and K equals 50 µm.

The dendritic morphology of the POU6F2-positive cells was characterized by staining the retinas of Thy1-YFP-H mice with antiserum directed against POU6F2. Approximately 3% of the RGCs in the Thy1-YFP-H mice are labeled with YFP, revealing the morphology of the cells including the dendritic arbors. In flat mounts, YFP labeled RGCs that co-stain with POU6F2 were identified and imaged to create a high-density z-stack (Fig. 1). The retinas were counterstained for ChAT to label ChAT-positive amacrine dendrites as a marker for sublaminae S2 and S4 of the inner plexiform layer. 3D reconstructions of the POU6F2-positive cells were created using Imaris software. The 3D reconstructions were then rotated 90° to identify the distribution of the ganglion cell dendrites in the inner plexiform layer (Fig. 1 D and E), allowing us to define the dendritic morphology of individual POU6F2 cells. We examined 10 retinas and found, total of 12 heavily labeled POU6F2 RGCs and 4 lightly labeled POU6F2 RGCs that were also marked by YFP. Of the 12 heavily labeled RGCs, all had dendrites that were bistratified in the inner plexiform layer, occupying sublaminae S2 and S4. Based on their dendritic morphology all of these cells were ooDSGCs. Furthermore, their dendritic expanse over the surface of the retina was consistent with the heavily labeled POU6F2 cells being directionally selective RGCs. The lightly labeled cells displayed differing laminar distribution. The cells had dendrites ramifying in the ON sublamina of the inner plexiform layer (n = 1) or in the OFF sublamina (n = 2) or in the ON–OFF sublaminae (n = 1). Thus, the majority (13 of 16) of the POU6F2-positive RGCs have dendrites ramifying in both the ON and OFF sublaminae of the inner plexiform layer whereas only lightly labeled POU6F2 RGCs have dendrites restricted to a single sublamina.

### Molecular characterization of POU6F2 RGCs

To define the POU6F2 RGC subtypes, we double-stained retinal flat-mounts for POU6F2 and second RGC-subtype specific markers. Retinas stained with secondary antibody only were used as controls. The markers we targeted are listed in Table 1. We co-stained retinas for POU6F2 with cell specific markers such as: SMI32 (alpha-RGC marker) and GAD67 and ChAT (amacrine cell markers). None of these markers shown to co-localized with POU6F2 (Table 1). Since the heavily labeled POU6F2 cells have dendrites ramifying in both the ON and OFF sublaminae of the inner plexiform layer, we examined markers for ooDSGC subtypes (Table 1). The first marker we examined was Cocaine- and Amphetamine-Regulated Transcript (CART), a marker for most known ooDSGCs in the mouse^13–16^. None of the POU6F2 cells were positive for CART. This was the first evidence that the POU6F2 cells may represent a novel subclass of RGC; previously most of the ooDSGCs were found to be CART positive^13^. We also stained retinas for SATB2, a known marker of ooDSGCs^17–19^, and again none of the POU6F2-positive cells were positive for this ooDSGC marker (Table 1). We used a reporter mouse line (B6.Cg-*Cdh6*^*tm1.1(cre/ERT2)Jrs*^/J, The Jackson Laboratory strain# 029428)^13,16^ to label Cdh6-positive cells with GFP. These retinas were then stained for POU6F2. Some of the RGCs were both POU6F2-positive and labeled with *Cdh6* driven GFP (Fig. 1F–H). Results showed that in double-labeled retinas, about 24% of the Cdh6-positive cells are also positive for POU6F2 (Fig. 1). The remaining 76% of the Cdh6 RGCS are POU6F2 negative. Hoxd10 also labels ooDSGCs in the mouse^20^. We used the Hoxd10-GFP reporter mouse that was introgressed onto the C57BL/6J strain to examine the co-labeling of Hoxd10 and POU6F2. When the retinas were stained for POU6F2, all of the Hoxd10 positive cells were also positive for POU6F2 (Fig. 1I-K). These cells represented approximately 3% of the heavily labeled POU6F2 cells; none of the lightly labeled POU6F2 RGCs were Hoxd10 positive. The total cells within the retina double labeled for Hoxd10 and POU6F2 was approximately 0.5% of the RGCs (213 cells/retina). These data demonstrate that the heavily labeled POU6F2 RGCs represent multiple RGC subtypes, with some labeled with Cdh6, others labeled with Hoxd10, and a population not labeled with the reporters for either Cdh6 or Hoxd10.

### Mouse strain differences in POU6F2 RGCs

We examined the distribution of POU6F2 positive RGCs in three strains of mice commonly used in vision research: C57BL/6J, DBA/2J, and BALB/c. In flat mounts of the retinas, cells were stained for POU6F2 and the pan-RGC marker RBPMS (Supplemental Fig. 1). This analysis revealed a significant difference in the proportion of POU6F2-positive RGCs in the different strains. The adult C57BL/6J retina had a total of 32.4% of the RGCs labeled POU6F2 positive, with 15.2% heavily labeled and 17.2% lightly labeled (Supplemental Fig. 1). A similar density of POU6F2 positive cells were observed in the DBA/2J mouse retina, exhibiting a total of 32.9% positive for POU6F2, with 16.1% being heavily labeled and 16.8% being lightly labeled (Supplemental Fig. 1). The examination of the BALB/c retina showed a higher proportion of RGCs labeled with POU6F2, having 64% of the RGCs labeled with POU6F2, with 27% being heavily labeled and 37% of the cells were lightly labeled. In all three strains, the POU6F2 cells were also labeled with RBPMS in the adult, indicating that POU6F2 only labels RGCs in the adult retina and the number of RGCs labeled by POU6F2 is dramatically different from strain to strain.

### Effects of glaucoma (DBA/2J mouse model)

**Figure 2:**
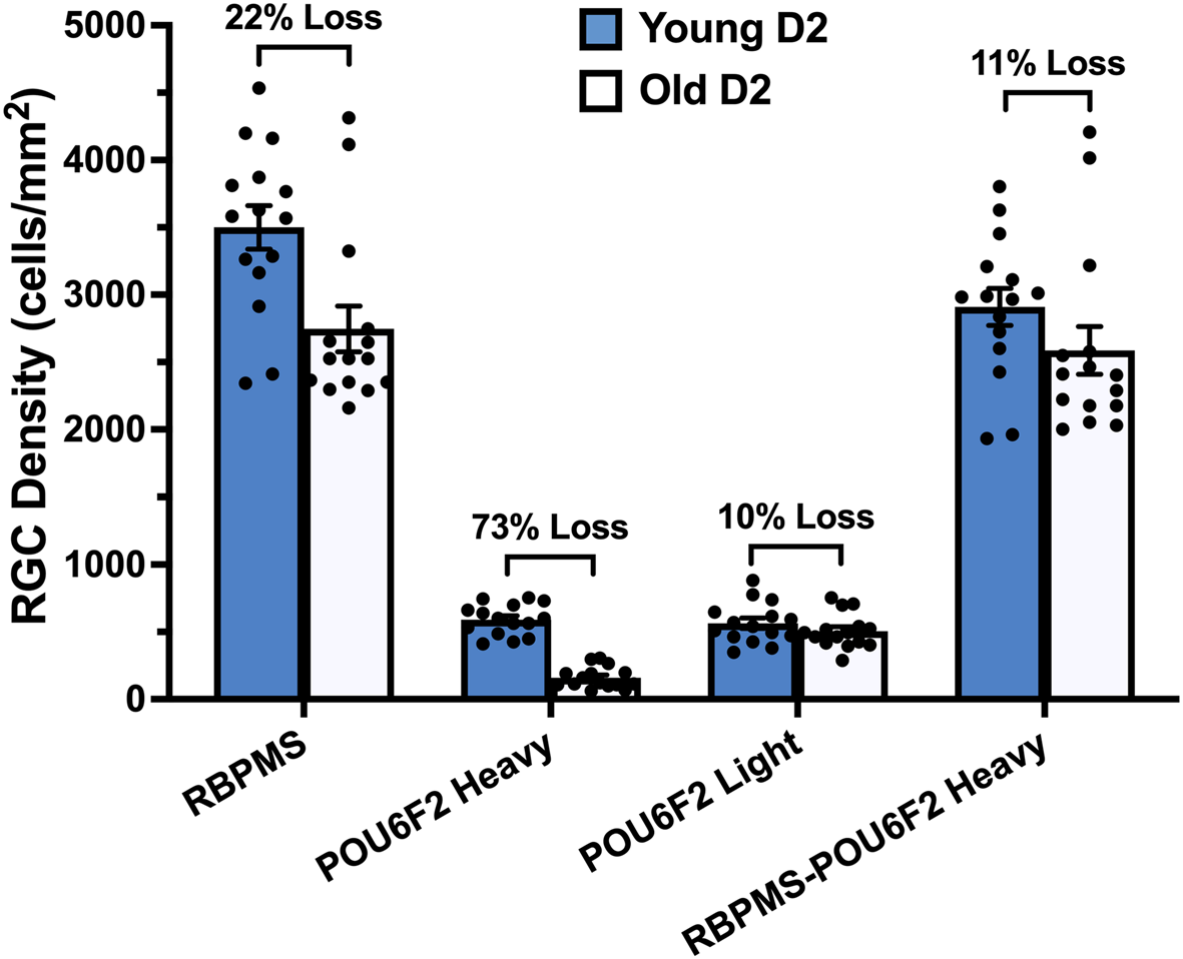
The selective sensitivity of the heavily labeled POU6F2 RGC subtypes was illustrated using the DBA/2J model of glaucoma. There was a 22% loss of RBPMS-labeled RGCs in aged DBA/2J mice (8 months of age, Old D2) as compared to young DBA/2J mice (2 months of age, Young D2). There was a dramatic loss (73%) of heavily labeled POU6F2-positive cells; while, there was a mild loss (10%) of the lightly labeled POU6F2 RGCs in the Old D2 mice when compared with Young D2. When we exclude the heavily labeled POU6F2-positive cells from the RBPMS labeled RGCs, there is an 11% loss of RGCs, approximately the same percentage of cell loss observed with the lightly labeled POU6F2-positive RGCs. Measures from individual animals are shown as dots. These data demonstrate the heavily labeled POU6F2 RGC subtypes is very susceptible to glaucomatous insult.

In our previous study^5^, we reported that the heavily labeled POU6F2 RGC subtype was very sensitive to early phases of glaucoma in the DBA/2J model^21^. We expanded this analysis to examine the distribution of both heavily labeled and lightly labeled POU6F2 RGCs. DBA/2J mice were examined at 8 months of age (n = 15), when disease progression includes early loss of RGCs. Control mice (n = 15) were 2 months of age, well prior to disease onset. There was a 22% loss of RPBMS-labeled RGCs and a 73% loss of POU6F2 heavily labeled RGCs in the aged DBA/2J mice (Fig. 2). When we examined the lightly labeled POU62 RGCs in the present study, there was only 10% loss of these cells (Fig. 2). If we examine the loss of RGCs labeled with RBPMS that are not also heavily labeled with POU6F2, there is an 11% loss of RBPMS-positive RGCs. This loss of RPBMS-positive minus the heavily labeled POU6F2 RGCs (11%) is approximately equal to the loss of lightly labeled POU6F2-labeled RGCs (10%) (Fig. 2). These data demonstrate that the heavily labelled POU6F2 RGCs were selectively more susceptible to glaucomatous injury in the early phases of glaucoma in the DBA/2J mouse model; while the lightly labeled POU6F2 RGCs have a similar susceptibility as other RGCs.

### Characterizing the ***Pou6f2***^−/−^ retina

**Figure 3:**
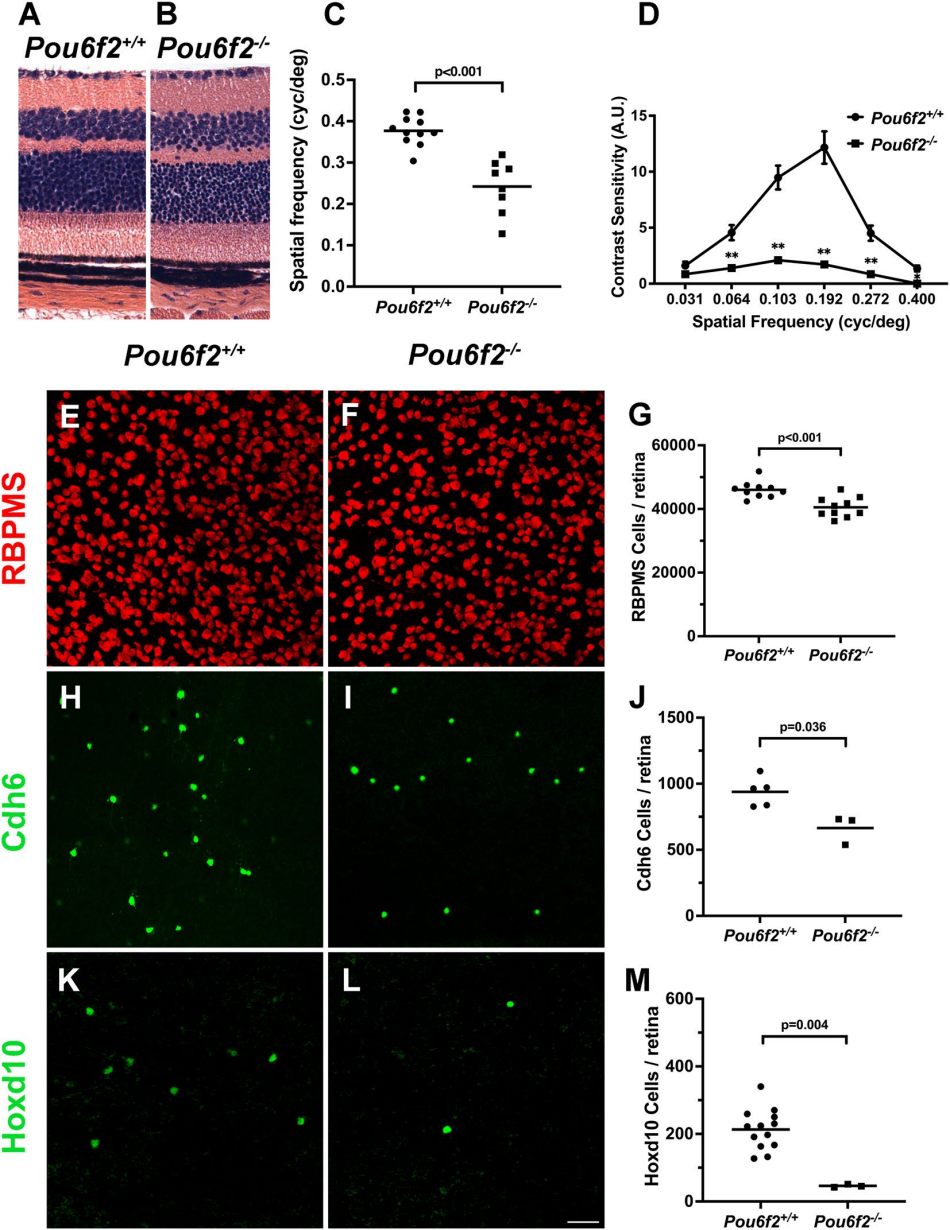
The effects of the *Pou6f2*^*−/−*^ mutation on the mouse. Sections through the *Pou6f2*^+*/*+^ retina (**A**) and the *Pou6f2*^*−/−*^ retina (**B**) strained with H and E reveal a similar morphology. The lamination of the retina is normal in both cases and the overall number of cells does not appear to differ. The visually guided behavior was examined using OMR to evaluate visual acuity (**C**) and contrast sensitivity (**D**) in WT mice (n = 11) and *Pou6f2*^−/−^ mice (n = 8). The *Pou6f2*^*−/−*^ had significantly lower spatial frequency detection than that of WT mice. Contrast sensitivity was measured at six different frequencies: 0.064, 0.103, 0.192, 0.272 and 0.400 cycles/degree. For contrast sensitivity the *Pou6f*^+*/*+^ mice displayed a normal inverted U-shaped curve; while in the *Pou6f2*^*−/−*^ mice the response was dramatically depressed. Notice the significant decrease in both visual acuity and contrast sensitivity. (Data presented with means ± SEM, Mann–Whitney U test, *p < 0.01, **p < 0.001). In the *Pou6f2*^*−/−*^ retina there is a loss of specific RGC subtypes. In retinas stained for RBPMS (**E** and **F**), there was on average a 11.4% loss of RGCs (**G**). This is similar to the total number of heavily labeled POU6F2 cells (15.2%). To determine if the POU6F2 cells were selectively missing from the knock out retina, the *Pou6F2*^*−/−*^ mutation was introgressed onto the *Cdh6*-CreER mouse (**H–J**) and the Hoxd10-YFP mouse (**K–M**). The data in **G**, **J**, and **M** are shown as means and the dots represent individual animals. When the retinas were examined, there was a 29.4% decrease (**J**) in the number of Cdh6 positive RGCs in the *Pou6F2*^*−/−*^ retinas relative the *Pou6F2*^+*/*+^ retinas. The results from the Hoxd10 mice were even more dramatic with an 81.3% decrease in Hoxd10-labeled RGCs in the *Pou6F2*^*−/−*^(**M**). The results were statistically significant using the Mann–Whitney U test. **E**, **F**, **H**, **I**, **K** and **L** were all taken at the same magnification and the scale bar in L represents 50 µm.

The effects of the *Pou6f2* germline null mutation on development of the retina was defined by comparing *Pou6f2*^+*/*+^ retinas to those of *Pou6f2*^+*/−*^ and *Pou6f2*^*−/−*^ mice (60 to 90 days of age). In H and E stained paraffin sections from *Pou6f2*^+*/*+^ (n = 15), *Pou6f2*^+*/−*^ (n = 18), and *Pou6f2*^*−/−*^ mice (n = 15), there was no apparent difference in the morphology of the retina (Fig. 3 A-B). All of the cellular layers were present and the thickness of the layers was similar. In addition, we could not detect any obvious differences in cell number or morphology within the H and E stained sections. To further examine RGCs in the *Pou6f2*^*−/−*^ mouse, flat mounts of the retinas from each of the genotypes, *Pou6f2*^+*/*+^ (n = 10), *Pou6f2*^+*/−*^ (n = 6) and *Pou6f2*^*−/−*^ (n = 10), were immunostained for RBPMS and POU6F2. The retinas were counterstained with TOPRO to label all nuclei. Each retina was coded by the PI (EEG) and all of the processing and analysis from this point on was conducted in a double-blind manner. Four fields were selected from each of the retinas and the total number of cells and the cells positive for RBPMS (RGCs) were counted. We calculated the number of RGCs/mm^2^ for each retina. The total number of RGCs was calculated using the total area of each retina. At the end of the counting period the codes were released and the mean number of RGCs and standard errors was calculated for each genotype. There was no difference in the number of RGCs between the *Pou6f2*^+/+^ and the *Pou6f2*^+/−^ group (Mann–Whitney U test). When we examined the *Pou6f2*^−/−^ retinas and compared them to the *Pou6f2*^+/+^ retinas, there were 11.4% fewer RGCs in the *Pou6f2*^−/−^ retinas (Fig. 3E–G). This difference is statistically significant (p < 0.001, Mann–Whitney U test). This is approximately equal to the number of heavily-labeled POU6F2 RGCs. To determine if a specific cell type was missing from the *Pou6f2*^*−/−*^ retina, we stained the retinas with three different RGC subtype specific markers and found no significant difference between the *Pou6f*2^−/−^ retinas and the wild type littermates. This included RGCs stained with SMI32 (a marker for alpha ganglion cells), CART (a marker for a class of ooDSGCs), and SATB2 (Supplemental Fig. 2). Thus, in the *Pou6f2*^*−/−*^ retina there is no loss of the subclasses of RGCs labeled these three subtype-specific RGC markers.

In the present study we found that a subpopulation of POU6F2-positive RGCs were also positive for Cdh6 or Hoxd10. To test for the potential loss of Cdh6 positive RGCs, the *Cdh6* reporter mouse line was placed on the *Pou6f2*^*−/−*^ background. For Cdh6, data were collected from *Pou6f2*^+*/*+^ retinas (n = 5) and *Pou6f2*^*−/−*^ retinas (n = 3). In the *Pou6f2*^+*/*+^ retina, the average number of Cdh6-positive RGCs was 940, with 24% being double labeled with POU6F2. In the *Pou6f2*^*−/−*^ retina 664 RGCs were labeled for Cdh6. Thus, there is approximately a 29.4% loss of Cdh6 RGCs in the *Pou6f2*^*−/−*^ retina (Fig. 3H–J) and the loss of Cdh6-positive RGCs was significant (Mann–Whitney U test, p = 0.036). These data indicate that the cells that are Cdh6 and POU6F2 positive appear to be missing from the *Pou6f2*^*−/−*^ retinas. The loss of Hoxd10-positive RGCs in the Pou6f2^−/−^ retina was even more dramatic than the reduction in this Cdh6 RGCs (Fig. 3 K–M). To examine the effects of the null-mutation on the Hoxd10 RGC subtype, the Hoxd10 reporter was introgressed onto the *Pou6f2*^*−/−*^ mouse strain. In the *Pou6f2*^*−/−*^ retina there were very few Hoxd10 positive cells with the average of 6 retinas being only 40 labeled RGCs per retina, while in the 13 wild type retinas the average number of Hoxd10 was 213 RGCs per retina and all of these cells are positive for POU6F2. This difference in Hoxd10 RGCs was statistically significant (Mann–Whitney U test, p = 0.001). Taken together, these data demonstrate that the cells missing from the *Pou6f2*^*−/−*^ retina are cells that would normally be heavily labeled POU6F2 RGCs some of which co-stained with Cdh6 or Hoxd10 (Table 1).

### Visual function in the ***Pou6f2***^−/−^ mouse

To investigate the effects of the *Pou6f2*-null mutation on visually-guided behavior, we examined visual acuity and contrast sensitivity of *Pou6f2*^+/+^ and *Pou6f2*^−/−^ mice using OptoMotry (OMR). The mice ranged in age from 200 to 250 days. For visual acuity, 11 *Pou6f2*^+/+^ mice and 8 *Pou6f2*^−/−^ mice were examined. The visual acuity of *Pou6f2*^−/−^ mice was much lower than that of WT mice. In the *Pou6f2*^−/−^ mice the acuity was measured to be 0.24 cyc/deg on average, while that in the *Pou6f2*^+/+^ mice it was 0.38 cyc/deg. This represents a significant decrease in visual acuity (p < 0.001, Mann–Whitney-U test, Fig. 3 C). We measured contrast sensitivity at six different spatial frequencies in 8 *Pou6f2*^+/+^ mice and 8 *Pou6f2*^−/−^ mice (Fig. 3 D). At low spatial frequencies (0.031 cyc/deg), no significant difference was detected between the two genotypes. As the spatial frequency increased, differences in response were observed, with the *Pou6f2*^−/−^ mice requiring higher contrast to respond to the grating. The typical inverted U-shaped contrast sensitivity function curve was observed in *Pou6f2*^+/+^ mice, while in the *Pou6f2*^−/−^ mouse, the response was reduced to an almost flat line. There was a gradual increase in the difference between *Pou6f2*^−/−^ mice and *Pou6f2*^+/+^ mice from 0.064 cyc/deg until the peak emerged at 0.192 cyc/deg, where the contrast sensitivity of *Pou6f2*^+/+^ mice was nearly 7 times higher than that of *Pou6f2*^−/−^ mice. At high spatial frequencies (0.272 and 0.400 cyc/deg), both groups presented downward trends, but the response of the *Pou6f2*^−/−^ mouse was decreased relative to the *Pou6f2*^+/+^ mouse. The magnitude of the OMR declined as the grating contrast decreased until reaching the contrast sensitivity threshold. As expected, none of the *Pou6f2*^−/−^ mice showed any response at 0.400 cyc/deg, while 5 out of 8 *Pou6f2*^+/+^ mice made up the final value, which was consistent with the visual acuity data. The differences were statistically significant (p < 0.001 at 0.064, 0.103, 0.192 and 0.272 cyc/deg, p = 0.008 at 0.400 cyc/deg, Mann–Whitney-U test). These data demonstrate that both visual acuity and contrast sensitivity as measured by OMR are severely affected by the *Pou6f2*^*−/−*^ mutation. Although this not direct proof of an effect of visual perception, it is suggestive of a deficit in visually guided behavior.

We recorded scotopic, full-field electroretinograms (ERG) to gain insight into retinal function of the *Pou6f2*-null mice. We examined 4 *Pou6f2*^*−/−*^ mice and 4 *Pou6f2*^+/+^ mice. There was no statistically detectable difference in the wave forms between the null and WT mice. There was no significant difference in mean a-wave amplitudes (*Pou6f2*^*−/−*^ mice had a Mean of −207uv with a Standard Deviation of 29uv and the *Pou6f2*^+/+^ mice had a Mean of −208uv with a Standard Deviation 12uv) or in mean b-wave amplitudes (*Pou6f2*^*−/−*^ mice had a Mean of 431uv with a Standard Deviation 57uv and for *Pou6f2*^+/+^ mice Mean 463uv with a Standard Deviation 14uv). Thus, the rod photoreceptors and bipolar cells of both null and *Pou6f2*^+/+^ mice appear to be functioning normally. To assess the function of RGCs in the *Pou6f2*^*−/−*^ mouse, pattern ERGs (pERGs) tests were conducted on 4 *Pou6f2*^*−/−*^mice, 4 *Pou6f2*^+*/−*^ and 4 *Pou6f2*^+/+^ littermates. The pERG waveforms were similar in all three groups (Supplemental Fig. 3). Quantitatively, there was no significant difference between any pERG measure. A similar finding was observed for the N2 peak with −15.67 for the *Pou6f2*^+/+^ mice, −18.29 for the heterozygote mice and −16.27 for the *Pou6f2*^*−/−*^ mice. Thus, the retina of the *Pou6f2*^*−/−*^ mouse functions normally within the bounds of scotopic ERG and pERG analyses.

### Monkey retina

**Figure 4:**
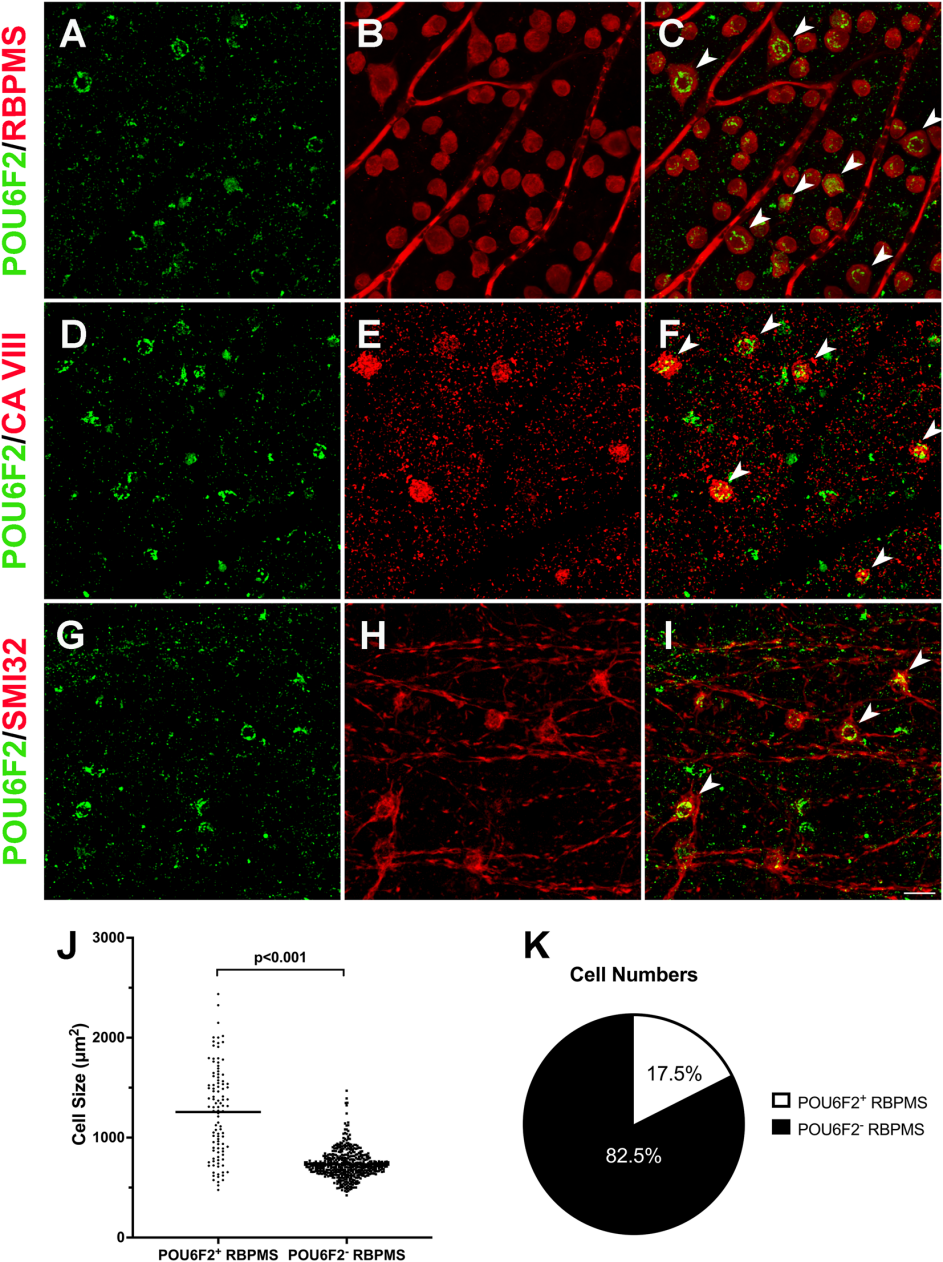
POU6F2 in the Macaque Retina. The rhesus macaque retina was stained for POU6F2 (**A**) and RBPMS (**B**) labeling a population of retinal ganglion cells. The merged image is in panel **C**. These cells appeared to be large parasol ganglion cells (arrow heads in **C**). To confirm this, retinas were stained for POU6F2 (**D**) and CAVIII (**E**), a marker for OFF parasol cells. The merged image is in panel **F**. All of the CAVIII cells were also positive for POU6F2 (arrow heads in **F**). To further identify these cells retinas were double labeled for POU6F2 (**G**) and neurofilament heavy chain (**H**) a known marker for parasol RGCs in the monkey. In the merged image (**I**) these neurofilament positive cells are also labeled with POU6F2. We examined the cell size of the POU6F2 cells in retinas counterstained for RBPMS (**J**). The size of individual cells is represented by dots. As can be seen in J the POU6F2-positive cells are larger than the POU6F2 negative RGCs. These POU6F2 RGCs make up 17.5% of the RGCs in the macaque retina (**K**). All photomicrographs were taken at the same magnification and the scale bar in I represents 50 µm.

The RGC subtypes in the mouse^7^ are considerably different from the RGC subtypes in non-human primates^22^ and human retina^23^. These differences make it difficult to define the relevance of our studies in the mouse to human biology. Since rhesus macaque RGCs are similar in class to those of the human, we stained macaque retinas for POU6F2. In retinas stained for POU6F2 and counterstained with RBPMS (a pan RGC marker) approximately 17.5% were double-labeled (Fig. 4). The mean diameter of the double-labeled cells was greater than that of cells the stained for POU6F2-negative RPBMS positive RGCs (Fig. 4). These large cells may be parasol RGCs, which are part of the magnocellular pathway. To confirm that these cells are indeed parasol RGCs, sections and flat mounts were stained for POU6F2 and counterstained for carbonic anhydrase 8 (CAVIII), a marker of OFF parasol RGCs^24^. In these sections, approximately half of the large POU6F2-positive RGCs were also labeled with CAVIII (Fig. 4 D-F), indicating that these cells are OFF-parasol RGCs. We assume that the remaining large POU6F2-positive cells are ON-parasol RGCs. To further define the cells expressing POU6F2, we stained macaque retina for POU6F2 and neurofilament heavy chain (a known marker of parasol cells in the macaque retina). In flat mounts and in sections of the retina, all of the cells labeled with the neurofilament heavy chain are also labeled with POU6F2 (Fig. 4 G-I). There were a few RGCs labeled with POU6F2 that were not neurofilament positive. Thus, in the non-human primate retina, POU6F2 labels the larger RGCs in the retina and most of these cells are parasol RGCs of the magnocellular pathway. This in general agrees with single cell RNA seq studies of human retina^23^.

## Discussion

The present paper identifies a group of RGCs in the mouse retina that express POU6F2. In the C57BL/6 mouse retina, 32.4% the RGCs express POU6F2, with 15.2% expressing high levels and 17.2% expressing moderate to low levels of the protein. We have focused on the RGCs expressing high levels of POU6F2 because these are the cells in the mouse that are relatively sensitive to glaucomatous injury. The results of the present study reveal that the heavily labeled POU6F2 RGCs are composed of multiple subpopulations, one labeled with Cdh6 and another labeled Hoxd10. These findings are in agreement with the single cell RNA seq. data from Tran et al.^7^. They found 6 RGC subtypes express high levels of *Pou6f2*. All six were classified as Novel: C7-Novel, C8-Novel, C10-Novel, C18-Novel, C37-Novel and C44-Novel. Based on our immunostaining, a subpopulation of these heavily labeled POU6F2 RGCs also express Cdh6. The data from Tran et al.^7^ reveals that three subtypes (C7-Novel, C8-Novel and C10-Novel), express both *Cdh6* and *Pou6f2*, whereas the other three subtypes (C18-Novel, C37-Novel and 44-Novel) do not express *Cdh6*. Furthermore, a small population (213 RGCs/retina) were labeled by the *Hoxd10* reporter gene.

To define the morphological class of the POU6F2 labeled cells, we examined the retinas of Thy1-YFP-H mice immunostained for POU6F2. All of the heavily labeled POU6F2 RGCs had dendrites that ramified in two sublaminae (S2 and S4) of the internal plexiform layer, indicating they were ON–OFF RGCs. Furthermore, their dendritic morphology was consistent with them being directionally selective. To identify the 12 POU6F2-positive RGCs in the Thy1-YFP-H mice in this study, we had to examine 10 flat-mount retinas. In each retina there were approximately 200 RGCs labeled with YFP and of these on average only one cell was found co-stained for POU6F2 in each retina. If these numbers were taken in isolation, one would believe that only 0.5% of the RGCs were heavily labeled POU6F2 RGCs. However, we know based on immunohistochemical staining of retina and data from single cell RNA seq studies^7^ that the number of heavily labeled cells is approximately 15.2% of the total RGCs. Thus, it appears that the labeling in the Thy1-YFP-H mouse is not random but has an inherent bias, and in our case against POU6F2-positive RGCs. To further illustrate the bias in the Thy1-YFP-H mouse retina we looked at a study by Bray et al.^25^. These authors found that the majority of the labeled RGCs of the Thy1-YFP-H mice were αRGCs (OSPN-positive, ~ 70%), while in single cell RNA seq studies^7^ only approximately 8% of the RGCs had an alpha RGC profile. Another example of potential bias comes from a study of directionally selective RGCs^13^ where the authors found that all of the ON–OFF cells were CART positive and represented 15% of the total RGCs. We have not observed double labeling of RGCs with CART and POU6F2 suggesting they are potentially two separate populations. We know that on the basis of RNA profiling^7^ these cells cell types represent multiple subclasses of RGCs. It is possible that specific subclasses of POU6F2 cells were not identified in the Thy1-YFP-H retina. Nonetheless, of the 12 heavily labeled POU6F2 cells reconstructed in this study all were ON–OFF directionally selective RGCs. These findings led us to explore two known markers of ooDSGCs, CART^13^ and SATB2^19^. Interestingly, none of the heavily labeled POU6F2 cells expressed either CART or SATB2. Thus, the heavily labeled POU6F2 RGCs represent a distinct subpopulation of ooDSGCs in the mouse retina. Our results indicate there are three types of ooDSGCs, one labeled by CART, one labeled by SATB2 and a group of RGCs positive for POU6F2.

### POU6F2 RGCs susceptible to injury

The heavily labeled POU6F2 cells are more susceptible to glaucomatous injury in the DBA/2J model. Previous studies demonstrate that the CART-positive ooDSGCs are susceptible to injury following optic nerve crush^26,27^. In these studies, it was thought that the retinal ooDSGCs were all CART positive^13,16, 26^. Based on our findings and those of Tran et al.^7^, the classification of ooDSGCs in the mouse suggests two groups of cells one CART positive and the other positive for POU6F2. The susceptibility of these cells to glaucomatous insult and optic nerve crush may differ between the two groups of cells. In this paper we demonstrate that 15.2% of the RGCs are heavily labeled POU6F2-positive ooDSGCs and these cells are not CART positive. Previous studies^13,26^ found that 15% of the RGCs are CART positive and they are ooDSGCs. Interestingly, the single cell RNA-seq study of the RGCs^7^ defined transcriptome changes of RGCs in response to injury, optic nerve crush. Their data showed that *Pou6f2* is one of the genes that is sensitive to injury and is down regulated within 12 h after optic nerve crush^7^. These findings support our observation that the POU6F2-positive cells are sensitive to glaucomatous injury in the DBA/2J mouse model of glaucoma^5^. This does not exclude the possibility that all ooDSGCs are in general the first to die after insult to the optic nerve.

In the present study we examined POU6F2 in the adult retina of three different mouse strains, finding that both C57BL/6J and DBA/2J mice had approximately the same proportion of POU6F2-positive RGCs, 32.4% for C57BL/6J and 32.9% for DBA/2J. The BALB/c mouse had significantly more POU6F2 positive cells, with 64% of the RGCs being positive for POU6F2. This strain-specific difference in the number of POU6F2-positive cells between the BALB/c retina and the retinas of the DBA/2J mouse and C57BL/6J mouse, may explain in part the differences observed in our previous study (32% POU6F2-positive RGCs)^5^ and the initial study from the Jeremy Nathans laboratory (50% to 60% POU6F2 positive RGCs)^1^. This type of strain difference was also observed for the Hoxd10 mouse strain. On the FVB background there were approximately 2000 RGCs expressing the marker protein GFP^20^; while, when this marker was introgressed onto the C57BL/6 background, only 213 RGCs per retina were observed expressing GFP. This difference could be due to inner retina remodeling or to differences in specific subtypes of RGCs due to the genetic background of different mouse.

When examining the retinal ganglion cells across different species the homology between different classes of RGCs is unclear. For most of the retinal cell types in the mammalian retina, the expression profiles are relatively constant across species^22^. This is not the case for RGCs^22^. We do know that POU6F2 is expressed in retinal ganglion cells in a number of different species, including: mouse^1,5^, cat^1^, monkey^1^ and human^23^. The presence of POU6F2 within a population of RGCs does not necessarily indicate that they are the same subpopulation of ganglion cells across different species. In the mouse there are at least 46 different types of RGCs and a large number of these cells are directionally selective^7^. While in the monkey and human the majority of the cells can be classified as magnocellular RGCs (10%) or parvocellular RGCs (80%). Furthermore, in the human directional selectivity is thought to be cortical. Numerous studies demonstrate directional selectivity in the primate visual cortex^28^ and there is very little evidence for directionally selective RGCs in the primate retina. A recent study found small percentage of macaque monkey RGCs are directionally selective^29^. Nonetheless the primate retina does not contain the high percentage of directionally selective RGCs as seen the mouse. POU6F2 does label specific RGCs in the macaque retina. They are the large parasol RGCs. When the retinas were counterstained for neurofilament, the cells were also labeled with POU6F2 demonstrating they were parasol RGCs. Some of these cells were also labeled with CAVIII a marker for OFF parasol RGCs. The molecular classifications of parasol cells in single cell RNA seq studies in macaque, marmoset and human demonstrate a strong conservation of key molecular components^22^.

Based on human GWAS results, POU6F2 is likely to contribute to multiple vision-based diseases. POU6F2 is a risk factor for glaucoma^8–10^, myopia^11^, and dyslexia^12^. It is difficult to directly relate our results in the mouse to these human diseases due to the considerable differences in RGC subtypes in the mouse and human. However, that we have shown that the POU6F2 RGCs are parasol cells in the macaque monkey allows for a direct interpretation of the potential role of POU6F2 in at least some of these diseases. In the mouse, the POU6F2 cells are more severely affected by glaucomatous injury. This also appears to be the case in primate glaucoma where the large cells are some of the first lost in experimental glaucoma^30^. These large axons are believed to come from the magnocellular (M) pathway or parasol cells that are neurofilament positive^31^. In chronic human glaucoma, the large diameter axons are missing from the optic nerve^32^, indicating that the human POU6F2-positive parasol cells are more severely affected by glaucomatous injury than other ganglion cell types. These parasol cells are part of the Magnocellular (M) pathway and psychophysiological testing indicates that the M-pathway is more severely affected in glaucoma^33^. Taken together, these data reveal that even though the cell types in the retina are different, ooDSGCs in the mouse versus parasol RGCs in the human, both are positive for POU6F2 and both are sensitive to glaucomatous injury.

Human GWAS reveals that POU6F2 is a risk factor for developing myopia^11^. Myopia is a complex trait with both genetic and environmental components affecting the structure of the eye, specifically the axial length of the eye. This leads to a defocused image on the retina. It appears that the control of axial elongation is intrinsic to the eye itself. In the monkey, monocular deprivation causes myopia and this myopia can occur even when the ciliary muscle is paralyzed or the optic nerve is sectioned^34^. When searching the Cell Atlas of the Human Fovea and Peripheral Retina (Single cell portal, Broad Institute; https://singlecell.broadinstitute.org/single_cell), POU6F2 is expressed only in RGCs with virtually no expression in any other cell type within the retina^23^. There is a modest expression of POU6F2 in the endothelial cells of the human cornea^35^, however, the most significant expression is in RGCs. We show that the parasol cells are expressing POU6F2. Thus, the data indicate the M-pathway is involved in the development of myopia. Interestingly, it is known that high myopes may have higher risk of glaucoma^36^ that may be due in part to shared genetic risk factors, such as POU6F2.

A case for the M-pathway (parasol RGCs) being involved in human dyslexia can also be made. In the *Pou6f2*^*−/−*^ mouse there is a loss of the heavily labeled POU6F2 RGCs and this leads to a dramatic depression of their contrast-sensitivity as measured by OMR. This may be similar to disruption of the parasol, magnocellular RGCs that occurs in dyslexia (for review see^37^). One of the deficits in most dyslexia cases is poor temporal processing and impaired magnocellular system processing^38^. The deficiency in the M-pathway in humans leads to decreased performance on frequency doubling illusion. The dyslexic subjects required an increase in contrast of the gradients as compared to non-affected subjects, indicating a decrease in ability to detect low contrast gratings. This may be related in part to the decrease in contrast sensitivity observed in the *Pou6f2*^*−/−*^ mouse observed in the present study. Some believe that this may be due to image stabilization in humans. This normally allows reading performance to be maintained while undergoing physical activity^39^. This is similar to previously proposed functional role of ooDSGCs in image stabilization in the mouse^20^.

## Conclusions

In the mouse the heavily labeled POU6F2 RGCs are ooDSGCs. while in the primate retina POU6F2 labels parasol RGCs. These POU6F2-positive cells are sensitive to damage in the DBA/2J mouse glaucoma model. In the *Pou6f2*^*−/−*^ mice there is a loss RGCs predominantly affecting the heavily labeled POU6F2-positive RGCs and this is associated with a significant loss of acuity and spatial contrast sensitivity as measured by OMR. In the primate retina, POU6F2 specifically labels the large parasol ganglion cells associated with the magnocellular (M) pathway. The association of POU6F2 with the M-pathway reveals its involvement human diseases, such as glaucoma, myopia, and dyslexia.

## Methods

Key resources table

**Table:** 

Reagent or resource	Dilution	Source	Identifier
Antibodies			
Rabbit anti-POU6F2	1:500	MyBiosource	Cat. #MBS9402684
Goat anti-ChAT	1:200	Millipore Sigma	Cat. #AB144P
Goat anti-CART	1:200	R&D System	Cat. #AF163
Mouse anti-SATB2	1:500	Abcam	Cat. #AB51502
Guinea pig anti-RBPMS	1:500	Millipore Sigma	Cat. #ARN1376
Mouse anti-TUJ1	1:1000	Gift from Anthony Frankfurter	N/A
Mouse anti-SMI32	1:500	BioLegend	Cat. #801701
Mouse anti-GAD67	1:1000	Millipore Sigma	Cat. #MAB4506
Chicken GFP	1:500	Millipore Sigma	Cat. #AB16901
Mouse anti-Carbonic Anhydrase VIII	1:500	NeoBiotechnologies	Cat. #767-MSM1-P1
Donkey anti-mouse, Alexa Fluor 488 conjugated	1:1000	Jackson Immunoresearch	Cat. #715-545-150
Donkey anti-rabbit, Alexa Fluor 488 conjugated	1:1000	Jackson Immunoresearch	Cat. #715-545-152
Donkey anti-rabbit, Alexa Fluor 594 conjugated	1:1000	Jackson Immunoresearch	Cat. #711-585-152
Donkey anti-rabbit, Alexa Fluor 647 conjugated	1:1000	Jackson Immunoresearch	Cat. #711-605-152
Donkey anti-chicken, Alexa Fluor 488 conjugated	1:1000	Jackson Immunoresearch	Cat. #703-545-155
Donkey anti-guinea pig, Alexa Fluor 594 conjugated	1:1000	Jackson Immunoresearch	Cat. #706-585-148
Donkey anti-guinea pig, Alexa Fluor 647 conjugated	1:1000	Jackson Immunoresearch	Cat. #706-605-148
Donkey anti-Goat, Alexa Fluor 647 conjugated	1:1000	Jackson Immunoresearch	Cat. #705-605-147
Donkey anti-Goat, Alexa Fluor 594 conjugated	1:1000	Jackson Immunoresearch	Cat. #705-585-147

Reagent or resource		Source	Identifier
*Chemicals, peptides, and recombinant proteins*			
Tamoxifen ≥ 99%		Sigma-Aldrich	Cat. #T5648-1G
Triton X-100		Sigma-Aldrich	Cat. #T9284-500ML
Tween-20		Fisher Bioreagents	Cat. #BP337-100
Donkey Serum		Sigma-Aldrich	Cat. #S30-100ML
TO-PRO-3		ThermoFisher Scientific	Cat. #T3605
Bovine Serum Albumin		Sigma® Life Science	Cat. #A9647-100G
Phosphate Buffered Saline 10X Molecular Biology Grade		Corning®	Cat. #46-013-CM
Ketamine		Vedco–KetaVed	Cat. #VINV-KETA-0VED
Xylazine		Akorn–AnaSed	Cat. # 59399011150
Antisedan		Zoetis	Cat. # 10000449
Paraformaldehyde 32% Solution		Electron Microscopy Sciences	Cat. #15714-S
Proparacaine Hydrochloride Ophthalmic Solution, USP 0.5% (Sterile)		Bausch & Lomb	Cat. #24208-730-06
Tropicamide Ophthalmic Solution, USP 1% Sterile		Akorn	Cat. #17478-102-12
VECTASHIELD Vibrance® Antifade Mounting Medium with DAPI		Vector Laboratories	Cat. #H-1800
Fluoromount-G®		Southern Biotech	Cat. #0100-01
Clear Nail Polish		Electron Microscopy Sciences	Cat. #72180
*Experimental Models: Organisms/Strains*			
Mouse: *C57BL/6J*		Jackson Laboratory	Strain #: 000664
Mouse: *BALB/c*		Jackson Laboratory	Strain #: 000651
Mouse: *DBA/2J*		Jackson Laboratory	Strain #: 000671
Mouse: Thy1-YFP-H^40^		Jackson Laboratory	Strain #: 003782
Mouse: *Hoxd10-GFP*		Mutant Mouse Resource and Research Center	Strain #: 032065-UCD
Mouse: *Pou6f2*^*−/−*^		Jackson Laboratory	Strain #: 009042
Mouse: *Hoxd10-GFP-Pou6f2*^*−/−*^^20^		Bred at Emory from strains listed above	N/A
Mouse: *Cdh6-CreER-zsGreen-Pou6f2*^*−/−*^^13^		Jackson Laboratory	Strain #: 029428
Rhesus macaque retina		Emory National Primate Research Center	N/A
*Software and Algorithms*			
Imaris		Oxford Instruments	N/A
Image J		NIH, USA	N/A
Photoshop		Adobe, USA	N/A
Cellprofiler		Broad Institute	N/A
Prism 9		https://www.graphpad.com/	N/A
SPSS 24.0		IBM	N/A
RGCode		https://gitlab.com/NCDRlab/rgcode	N/A
Human Cell Atlas		Broad Institute	N/A
*Other*			
24-well plates		Corning	Cat. #3524
96-well plates		VWR INTERNATIONAL INC	Cat. # 41-12-21-02
VWR micro cover glass		VWR INTERNATIONAL INC	Cat. #48366-227
VWR micro slides		VWR INTERNATIONAL INC	Cat. #48311-703
Micro dissecting forceps-Straight		Roboz Surgical Store	Cat. #RS-5070
Micro dissecting forceps-Angled		Roboz Surgical Store	Cat. #RS-5095
Vannas Spring Scissors-Straight		FINE SCIENCE TOOLS	Cat. #150000-08
Optomotor Response		Cerebral Mechanics Inc	Model: OptoMotry
Electroretinograms		Diagnosys	Model #Celeris D432

## Contact for reagent and resource sharing

Further information and requests for resources and reagents should be directed to and will be fulfilled by the Lead Contact, Eldon Geisert (egeiser@emory.edu).

## Experimental model and subject details

Ten Thy1-YFP-H mice (B6.Cg-Tg(Thy1-YFPHJrs/J, Stock#:003782)), and the Pou6f2^−/−^ mouse strain (B6.129-*Pou6f2*^*tm1Nat*^/J, Strain #: 009042) were purchased from Jackson Laboratory (Bar Harbor, ME, USA) (n = 25). In the Thy1-YFP-H mouse line approximately 3–10% of the RGCs are labeled by YFP in the retina^41^. This allows us to identify the morphology of RGCs that were positive for the marker POU6F2. We also stained retinas from *Cdh6*-CreER mice (n = 5), B6.Cg-*Cdh6*^*tm1.1(cre/ERT2)Jrs*^/J, Jax Stock No:029428)^42^ to determine if there were independent markers for the POU6F2 cells identifying the specific POU6F2-positive ganglion cell subtype^43^. The Hoxd10 reporter mice were acquired from the Mutant Mouse Resource and Research Center. The *Hoxd10* marker was introgressed from the FVB/NTac strain to the C57BL/6J background. The DBA/2J mice were purchased from The Jackson Laboratories (Stock No:000671). To study the effects of glaucoma on retinal ganglion cell subtypes we used the DBA/2J model comparing aged DBA/2J mice (8 months old) to young adult DBA/2J mice (2 months old). We also studied the distribution of POU6F2-positive cells in C57BL/6J mice (n = 4 per group) and BALB/c mice (n = 4 per group). Rhesus Macaque retinas (n = 2) were obtained from the Emory National Primate Research Center.

All procedures involving animals were approved by the Animal Care and Use Committee of Emory University and were in accordance with the ARVO Statement for the Use of Animals in Ophthalmic and Vision Research. The mice were housed in a pathogen-free facility at Emory University, maintained on a 12 h:12 h light–dark cycle, and provided with food and water ad libitum.

## Method details

### Immunohistochemistry

For immunohistochemistry, the mice were deeply anesthetized with intraperitoneal injections of 100 mg/kg ketamine and 10 mg/kg xylazine, and perfused through the heart with saline followed by 4% paraformaldehyde in phosphate buffer (pH 7.3). The eyes were removed, and the retinas were dissected out. Flat-mounts of the retina were prepared for staining using a protocol similar to that previously described^5^. The retinas were stained for primary antibodies followed by fluorescent-tagged secondary antibodies. All antibodies and the dilutions used are listed in Key Resource Table. The retinas were blocked in 3% BSA and 3% donkey serum in 0.5% Triton X-100 in PBS. After the primary antibodies staining for over two nights at 4 °C, the retinas were rinsed three times in PBS with Tween-20 (0.1%) at room temperature. The retinas or sections were then placed in secondary antibodies which included AlexaFluor 488 AffiniPure Donkey Anti-Rabbit, Cat. #715-545-152 or Alexa Fluor 594 AffiniPure Donkey Anti-Guinea pig, Cat. #706-585-148 (Jackson Immunoresearch, West Grove). For molecular characterization of the POU6F2-positive RGCs, the retinas were double stained for POU6F2 and a second antibody to identify specific retinal cell subtypes in the ganglion cell layer (Key Resources Table). The macaque retinas were stained with a mouse monoclonal antibody directed against carbonic anhydrase VIII (NeoBiotechnologies Union City CA) to identify OFF parasol RGCs, SMI32 to label all parasol RGCs and RBPMS to define total RGCs. Selected retinal flat-mounts were also stained for TO-PRO-3 to label nuclei in the retina.

### Image processing

For 3D reconstructions of POU6F2-positive RGCs in the Thy1-YFP-H mouse, the retinal flat-mounts were imaged using a Nikon Eclipse Ti2 (Nikon, Inc., Melville, NY, United States) microscope with A1R confocal imager. The RGCs double labeled for POU6F2 and YFP were scanned under 40X magnification. Z-stacked images were taken at 0.1 μm increments, with a total of 300–600 optical slices for each retina including all dendrites. 3D reconstructions of the POU6F2-positive cells were made from z-stacks of confocal images using the Imaris software (v9.2, Bitplane, Oxford Instruments, Switzerland). The 3D reconstructions were then rotated 90° to identify the distribution of the ganglion cell dendrites. These reconstructions revealed the general morphology of the labeled RGCs as well as the localization of the dendrites in the internal plexiform layer, identifying ON-RGCs, OFF-RGCs and ON/OFF RGCs.

To quantify the number of RGCs labeled with each of the subtype-specific markers, retinal flat-mounts were image on a Nikon Ti2 confocal microscope with A1R confocal imager (Nikon, Inc., Melville, NY, United States). For each retina, the total number of labeled RGCs for each retina was determined using CellProfiler (v3.1.5)^44,45^ and RGCode^46^. The pipeline follows the method from Dordea et al.^47^ with modifications to optimize the counting for POU6F2 and RPBMS positive cells. For each RGC marker, at least 3 independent biological retina samples were examined for the quantification of RGCs. Immunostained sections and H and E stained sections were photographed using either the Nikon Ti2 confocal microscope with a DS-Ri2 camera (Nikon, Inc., Melville, NY, United States) or an Olympus BX51 microscope and a Microfire camera (Optronix).

### Optomotor response (OMR)-based visual assessment

Real time video tracking and visually evoked compensatory head movements measurements were performed by an OMR recording setup (OptoMotry, Cerebral Mechanics Inc., Lethbridge, Alberta, Canada). Four computer-controlled identical, connected 17-inch^48^ LCD screens form the display monitors. During measurements, stimuli consisting of black and white striped patterns with distinct luminosities, contrasts, spatial, or temporal frequencies can be presented in a sequential or step-wise manner^49^. The optomotor stimulus consisted of vertical sine-wave gratings that rotated at a constant speed (12°/s)^50^, and were presented randomly in either clockwise or counterclockwise direction. A 5 cm-diameter platform was set in the middle of the arena for mouse to stand and move freely on. An observer blinded to the identity of the subject mouse tracked the middle of the mouse head manually to dynamically readjust the actual physical distance to the cylinder, which guarantees that the animal perceives a constant grating when the head is moving^51^. The ability of mice to detect and react to the stimulus by head/body movements is judged for each individual subject by the same observer who was blinded to the identity of the subject mouse. A minimum of three instances of head tracking observed was required to conclude that the animal could detect the stimulus. All the OMR experiments were run double-blind. Animals were coded with a number and the codes were given maintained by independent lab member (either EEG or SJ). The experiment was carried out by a blinded observer (either BK, YL or FL). After the experiments were completed, the codes were released and the data was compiled.

Visual acuity has been defined as the highest spatial frequency that the mouse could track in either direction^48^. Using the staircase procedure^52^, spatial frequency was systematically increased after successive trails until the mouse no longer responded, while the sinusoidal gratings were all at 100% contrast. CS was testing at a fixed spatial frequency began with a grating of 100% contrast, which was then systematically reduced until the contrast threshold was identified. The threshold was calculated as a Michelson contrast from the screen’s luminance (maximum − minimum)/(maximum + minimum)^48^. The contrast sensitivity plotted on the graph was the reciprocal of the threshold. In addition, a contrast threshold was identified in our study at six spatial frequencies (0.031, 0.064, 0.103, 0.192, 0.272, 0.400 cyc/deg).

### Electroretinograms (ERGs)

The ERG protocol was previously described in detail^53^. Briefly, 4 *Pou6f2*^*−/−*^ and 4 *Pou6f2*^+*/*+^ mice dark-adapted overnight and experiments were conducted in dim red light. Mouse were anesthetized with 100 mg/kg ketamine and 10 mg/kg xylazine, followed by 0.5% Proparacaine eye drops as local anesthesia. Pupils were dilated with 1% Tropicamide for 5 miniutes. Each mouce was placed on a heating pad (39 °C) under dim red light provided by the overhead lamp of the Diagnosys Celeris ERG apparatus (Diagnosys, LLC, Lowell, MA, USA). Individual eyes had light-guided electrodes placed in contact with them, with the corneal electrode of contralateral eye serving as the reference. We recorded full-field ERGs under scotopic conditions using varying stimulus intensities (0.001, 0.005, 0.01, 0.1, and 1 cd s/m^2^) with a flash duration of 4 ms. Signals were captured for 0.3 s after each step to assess scotopic a- and b-wave function. Following the recordings, each mouse was placed in its home cage on a heating pad (39 °C) to aid recovery from anesthesia.

Pattern electroretinograms (pERG) was used to assess RGC function (Supplemental Fig. 3). The amplitudes of the P1 and N2 components were measured. P1 amplitude was measured from the N1 nadir to the peak of P1. N2 amplitude was measured from the preceding P1 peak to the nadir of N2 or “P1N2”. After being dark-adapted overnight the mice were anesthetized as previously described, and local anesthesia eyedrop proparacaine was used prior to pupil dilation eyedrop Tropicamide. Mice were placed under dim red light from the overhead lamp of the Celeris-Diagnosys system (Diagnosys, LLC, Lowell, MA, USA). The pattern stimulator was placed in contact with the eye; the flash stimulator for the contralateral eye acted as the reference electrode. Transient pERG responses were recorded using black and white vertical stimuli delivered on the Celeris system using manufacturer’s guidelines. Briefly, pattern stimuli of 50 cd·s/m^2^ were presented and 600 averaged signals with cut-off filter frequencies of 1 to 300 Hz were recorded under scotopic conditions.

### Statistical analysis

Data are presented as mean ± standard error of the mean (SEM). Differences in RGC counts were analyzed with the Mann–Whitney U-test using SPSS statistics package 24.0 (SPSS, IBM, Chicago, IL, United States). A *p*-value of less than 0.05 was considered statistically significant.

### Supplementary Information

Supplementary Figures.

## Data Availability

The data used and/or analyzed during the current study are available from the corresponding author on reasonable request.
