# Supplementary material for: POU6F2, a risk factor for glaucoma, myopia and dyslexia, labels specific populations of retinal ganglion cells
Source: Sci Rep. 2024 May 2;14:10096. doi: 10.1038/s41598-024-60444-5 (PMC11066091; doi:10.1038/s41598-024-60444-5)
Supplement: Supplementary file 1 — Supplementary Figures. [file 41598_2024_60444_MOESM1_ESM.docx]

**POU6F2, a Risk Factor for Glaucoma, Myopia and Dyslexia, Labels Specific Populations of Retinal Ganglion Cells**

Fangyu Lin^1*^, Ying Li^1*^, Jiaxing Wang^1^, Sandra Jardines^1**^, Rebecca King^1^, Micah A. Chrenek^1^, Janey L. Wiggs^2^, Jeffrey H. Boatright^13^, and Eldon E. Geisert^1***^

^1^ Department of Ophthalmology, Emory University, 1365B Clifton Road NE Atlanta GA, 30322, USA.

^2^Massachusetts Eye and Ear, Harvard Medical School Boston, Boston, MA, USA.

^3^ Atlanta Veterans Administration Center for Visual and Neurocognitive Rehabilitation, Decatur, Georgia, USA

* Fangyu Lin and Ying Li are co-first authors.

^**^ Current Address: Icahn School of Medicine at Mount Sinai, 1 Gustave L. Levy Pl, New York, New York 10029. [sandra.jardines@icahn.mssm.edu](mailto:sandra.jardines@icahn.mssm.edu)

***Corresponding Author: Eldon E. Geisert

Professor of Ophthalmology

Emory University

1365B Clifton Road NE

Atlanta GA 30322

email: egeiser@emory.edu

Phone: 404-778-4239

**Supplemental Figures**


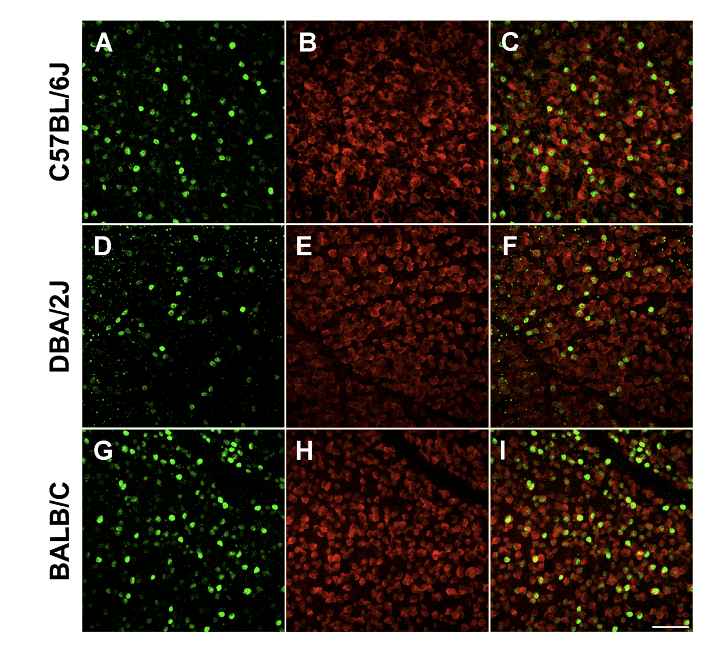


**Supplemental Figure 1:** Strain differences in the number of POU6F2 positive RGCs. RGCs were stained for POU6F2 (Green) and RBPMS (Red) in retinas from 3 different strains of mice: C57BL/6J (A-C), DBA/2J (D-F) and BALB/c (G-I). Notice that the number of POU6F2 positive cells are approximately equal in the C57BL/6J (A-C), DBA/2J (D-F) retinas; while there are significantly more in the BALB/c retina (G-I). Scale bar in I represents 50µm.


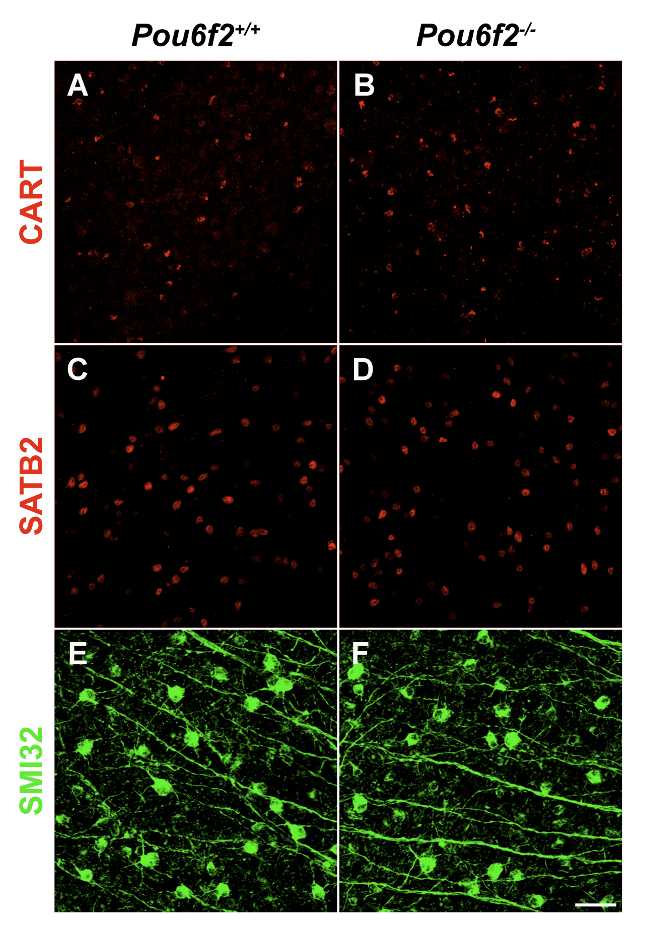


**Supplemental Figure 2:** RGC subtypes not lost in the *Pou6f2^-/-^* mouse. Retinas from the *Pou6f2^+/+^* and *Pou6f2^-/-^* were stained for three different markers for RGC subtypes: CART (A, B), SATB2 (C, D) and SMI32 (E, F). The number of each of these RGC subtypes was similar between the *Pou6f2^+/+^* (A, C, and E) and *Pou6f2^-/-^* (B, D and F) retinas. All photomicrographs are taken at the same magnification and the scale bar in F represents 50 µm.


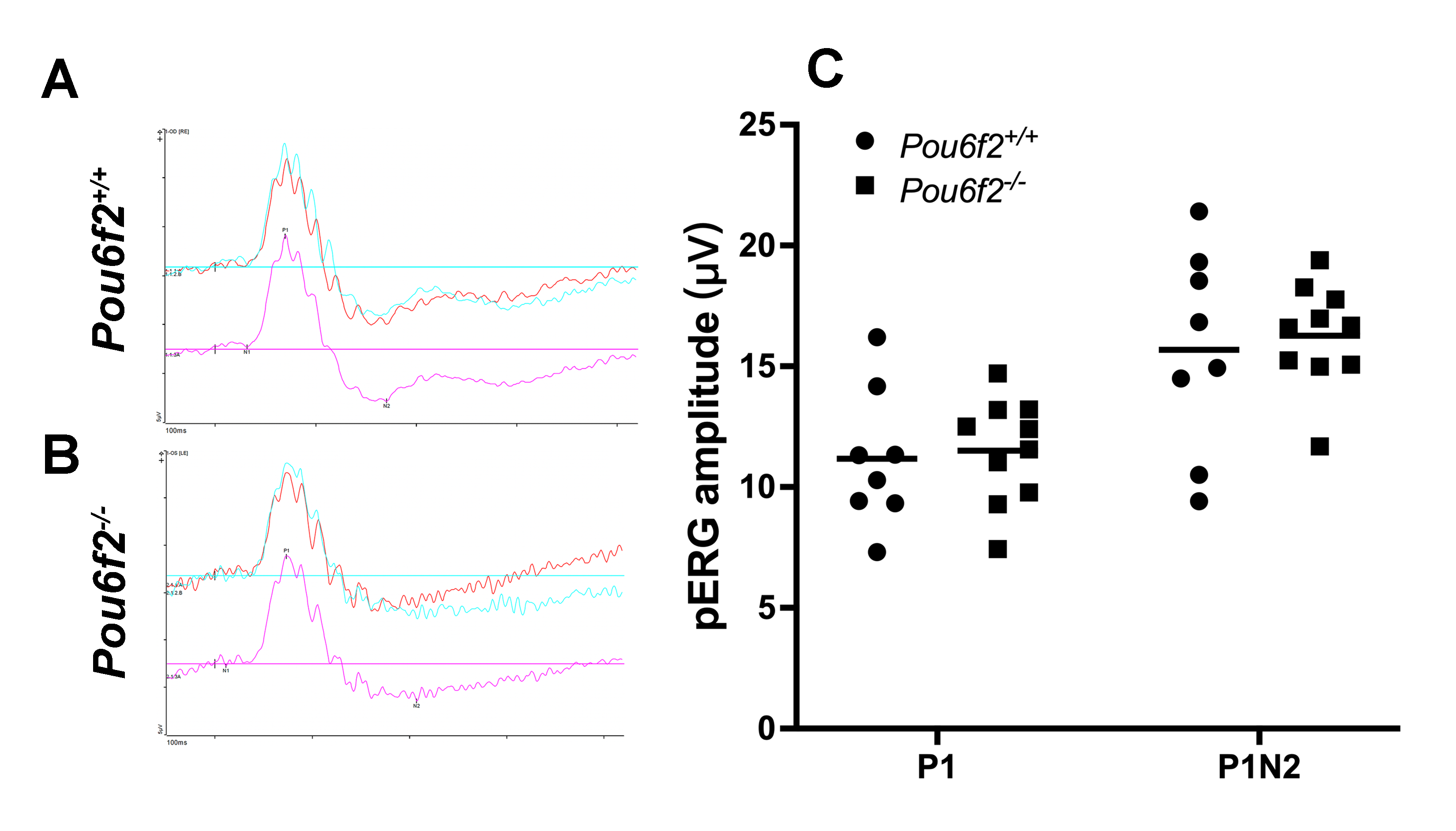


**Supplemental Figure 3:** Patterned ERGs (pERGs) were taken from *Pou6f2^+/+^* and *Pou6f2^-/-^* mice. Examples of the tracings are shown in panels A and B. The tracing does not show any significant difference between the *Pou6f2^+/+^* and *Pou6f2^-/-^* mice. When examining P1 there is no significant difference nor is there any significant difference in P1N2 (C). Measures from individual animals are shown as dots or squares. These data demonstrate that the retinas of the *Pou6f2^-/-^* mice are functioning. The red and blue traces are two different tests, and pink is the average of the two.
